# Data-as-a-Service Platform for Delivering Healthy Lifestyle and Preventive Medicine: Concept and Structure of the DAPHNE Project

**DOI:** 10.2196/resprot.6589

**Published:** 2016-12-09

**Authors:** Catherine Gibbons, Gonzalo Bailador del Pozo, Javier Andrés, Tim Lobstein, Melania Manco, Hadas Lewy, Einat Bergman, David O'Callaghan, Gavin Doherty, Olga Kudrautseva, Angel Palomares, Roni Ram, Alberto Olmo

**Affiliations:** ^1^ School of Psychology, University of Leeds Leeds United Kingdom; ^2^ CeDInt Technical University of Madrid Madrid Spain; ^3^ World Obesity Federation London United Kingdom; ^4^ Bambino Gesù Children's Hospital Rome Italy; ^5^ Nevet Ltd Tel-Aviv Israel; ^6^ SilverCloud Health Ltd Dublin Ireland; ^7^ Atos Research and Innovation Madrid Spain; ^8^ IBM Research Haifa Israel; ^9^ Treelogic SL Madrid Spain

**Keywords:** information communications technology, clinical weight management, physician and patient portal, obesity, physical activity, advanced motion sensors, behavior change

## Abstract

**Background:**

Overweight and obesity is related to many health problems and diseases. The current obesity epidemic, which is a major health problem, is closely related to a lack of physical activity, high levels of sedentary behavior, and increased energy intake; with evidence to show increasing incidence of these issues in the younger population. Tackling obesity and its comorbid conditions requires a holistic approach encompassing attention on physical activity, healthy diet, and behavioral activation in order to enable and maintain meaningful and long-term weight loss and weight maintenance.

**Objective:**

The objective of the Data-as-a-Service Platform for Healthy Lifestyle and Preventive Medicine (DAPHNE) project is to develop a breakthrough information communications technology (ICT) platform for tracking health, weight, physical activity, diet, lifestyle, and psychological components within health care systems, whereby the platform and clinical support is linked.

**Methods:**

The DAPHNE platform aims to deliver personalized guidance services for lifestyle management to the citizen/patient by means of (1) advanced sensors and mobile phone apps to acquire and store continuous/real-time data on lifestyle aspects, behavior, and surrounding environment; (2) individual models to monitor their health and fitness status; (3) intelligent data processing for the recognition of behavioral trends; and (4) specific services for personalized guidance on healthy lifestyle and disease prevention. It is well known that weight loss and maintenance of weight loss are particularly difficult. This tool will address some of the issues found with conventional treatment/advice in that it will collect data in real time, thereby reducing reliability issues known with recalling events once they have passed and will also allow adjustment of behavior through timely support and recommendations sent through the platform without the necessity of formal one-to-one visits between patient and clinician. Patient motivation/compliance is a particular issue with conventional weight loss regimes; DAPHNE aims to increase the individuals’ awareness of their own behavior and fosters their accountability.

**Results:**

The project has been funded and the research work has started. Results for the validation of the different components is due imminently.

**Conclusions:**

In contrast with previous existing solutions, the DAPHNE project tackles the obesity problem from a clinical point of view, designing the different interfaces for its use by patients (adults and children), physicians, and caregivers. A specific design for children and adolescent patients treated for obesity has been followed, guided by pediatric physicians at hospitals in Europe. The final clinical validation of the DAPHNE platform will be carried out in different European hospitals, testing the platform in both adolescents and adults.

## Introduction

### The DAPHNE Concept

The prevalence of overweight and obesity across Europe is high, with rates doubling during the last few decades in several countries. More than 50% of the total European adult population is now overweight (body mass index [BMI]>25 kg/m^2^) and obesity levels (BMI>30 kg/m^2^) of adults in many Member States on average exceed 20% [1]. Data from several studies suggest that childhood obesity has also increased steadily in Europe over the past two to three decades. In Europe, almost 20% of children are overweight or obese [2]. The highest prevalence levels are observed in southern European countries [3]. Both in young and adult populations, obesity is now therefore regarded as one of the most important determinants of avoidable burden of disease. From our knowledge, there are 2 commercially available products similar to the Data-as-a-Service Platform for Healthy Lifestyle and Preventive Medicine (DAPHNE) concept. “Retrofit” [4] is the provider of corporate weight loss programs and “Omada” [5] is designed to help individuals lose weight. Both are largely aimed at behavior change and the combination of exercise physiologists and dietitians. These 2 products have been successful and a number of characteristics are similar to those proposed by DAPHNE. However, the major difference is that DAPHNE is being designed as a system to be used between a physician/caregiver and the patient having taken into account the privacy and security aspects of this scenario.

### The DAPHNE Approach

A holistic approach is required when investigating obesity, both energy expenditure and energy intake are important. There is irrefutable evidence of the effectiveness of regular physical activity in the primary (preventing disease in the first place) and secondary prevention (halting/slowing the progress of disease) of several chronic diseases (eg, cardiovascular disease [CVD], diabetes, cancer, hypertension, obesity, and osteoporosis) [6], and the more activity people partake in the more the risks of ill-health are reduced [7]. There is little doubt in the adult population that high levels of physical activity are associated with reduced risk of type 2 diabetes, CVD, and premature mortality [8-10]. There have been some longitudinal studies examining the effect of physical activity and fitness during the early years on CVD risk later in life. Overall, these studies agree that high levels of physical fitness during adolescence and young adulthood are related to healthy CVD risk profiles later in life; however, physical activity appears to have little influence later in life. Steinbeck et al [11] have shown cross-sectional evidence linking physical inactivity to the development of obesity in children, but as of yet there is little evidence showing physical inactivity preceding the weight gain. Exercise is likely to be most effective in controlling childhood obesity when it is combined with appropriate dietary changes [12]. Furthermore, there are a number of diets advocated in the literature to be successful for weight loss and weight maintenance [13,14]. Overall, the Mediterranean diet is often seen as the best diet to follow at population level [15,16]. All diets have an element of control, whether it be low fat, low carbohydrate, at least 5 pieces of fruit and vegetables, and so on. Governmental agencies across Europe have guidelines for dietary advice in place and it will be these that are followed within the DAPHNE project [15,16].

In addition to the metabolic and physiological benefits of exercise and physical activity, there is growing evidence that exercise can be effective in improving the mental wellbeing of the general public, largely through improved mood and self-perceptions. These are often major concerns in those with weight or obesity problems. Because the concept of the DAPHNE project is a holistic approach, we have decided to add the functionality to measure psychological wellbeing. It is generally assumed that exercise also exerts a positive effect on psychological functioning (ie, reduction in symptoms of depression, anxiety, stress, and negative mood states) and previous studies corroborate this assumption. It has been demonstrated that there is an inverse association between physical activity and likelihood of depression, mainly considering higher levels of physical activity (as recommended in physical activity guidelines), but also at lower doses [17,18]. There is evidence suggesting positive effects of exercise on depression in intervention [19,20], cross-sectional [21], population-based [22], and cohort studies [23-25], indicating that participation in exercise could be an important target of mental health treatments. Further to this, exercise has been shown to be effective as a treatment for clinical depression [26] and anxiety in both adults [27] and adolescents [28].

Both energy expenditure through exercise/physical activity and energy intake through food consumption are deemed important in the control of energy balance. There is a plethora of studies looking at the role of diet in obesity and advice doctors provide is typically based on the country’s policy in this area. Specific dietary guidelines of less fat, more fruit and vegetables, and so on are commonplace; the DAPHNE system and mobile apps will be built with these standards. Therefore, the aim of the current project is to design and develop information communications technology (ICT) platforms that will enable the monitoring, intervention, and follow-up of health behavior using measurement and tracking of both energy expenditure and energy intake, along with anthropometric variables, health markers, and psychological status. Furthermore, a secondary aim of this project is to design the platforms for clinical use and to improve the link between physicians (and their care team of nutritionists, physiotherapists, etc) and their patients and have an additive effect to usual treatment.

The last decade has seen growing popularity and uptake of self-monitoring technology including wireless sensor devices and mobile apps for tracking physical activity, sedentary behavior, sleep patterns, diet, and stress. However, their application as a means of monitoring and motivating out patients in health service settings remains to be developed and the application and interaction between the care provider and the patient using innovative ICT tools for health behavior change is limited, and there is currently no system that enables involvement of both parties in the process of preventive medicine. Furthermore, the system will be developed for use by both adolescents and adults with input of physicians regarding the different requirements of both population groups. DAPHNE is a collaborative European research project. The objective of the project is to develop an innovative ICT platform toward a holistic approach for weight management through measurement and tracking of physical activity, diet, lifestyle, health, and weight over time and validate the use of this platform in clinical settings, assessing the acceptability of the proposed solutions and the benefits for physicians and patients. The present manuscript aims to identify and explain the structure of the DAPHNE platform including full details about the data input and output that will be provided.

## Methods

### Overview of the Architecture

**Figure 1:**
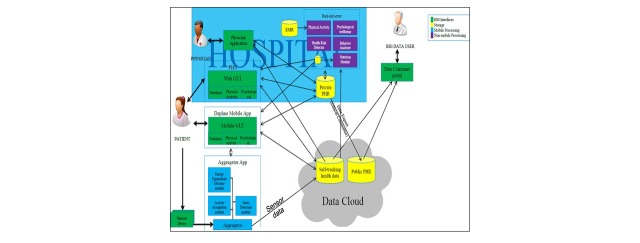
Overview of Data-as-a-Service Platform for Healthy Lifestyle and Preventive Medicine (DAPHNE) architecture.

The objective of the DAPHNE project is to provide the services for patients within health care systems, but also for the wellbeing of users who would not be linked to a health care physician. For the purpose of this manuscript, only the version developed for the health care system will be discussed because it is, to our knowledge, the only model to do so (Figure 1). The Personal Health Services (PHS) of the DAPHNE project are provided through 3 pathways: the PHS portal, the mobile phone apps, and the physician application in order to assist in the provision of continuous, quality-controlled, PHS (Textbox 1). These services can be implemented in a wide variety of environments, such as hospitals, clinics, sports teams, and ultimately, in any environments that have external health advisers/trainers/coaches and/or end users that need or decide to lead a healthy lifestyle.

**How the developed services are provided.**

**Figure 2:**
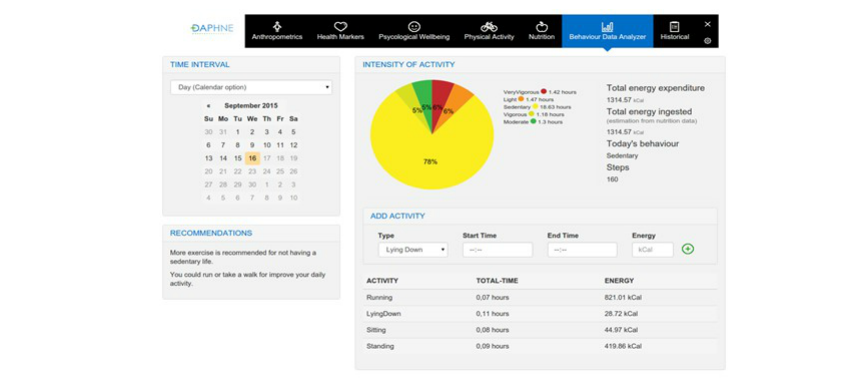
Screenshot of the Data-as-a-Service Platform for Healthy Lifestyle and Preventive Medicine (DAPHNE) personal health system.

1. Personal Health Services (PHS) Portal:

This is the access point for patients and caregivers (Figure 2). It consists of a Web-based app where users can check and see their information in the platform. This tool allows users to control their health information, motion sensor analysis, and receive personalized recommendations. In order to facilitate the usability of the portal and aid the insertion of data, some PHS services will be based on information provided or collected through mobile phone apps.

2. Data-as-a-Service Platform for Healthy Lifestyle and Preventive Medicine (DAPHNE) mobile phone apps:

These apps allow users to enter their nutrition data (via mobile and Web interfaces, connected to the European Food Information Resource (EuroFIR) [29] nutritional database) and monitor their physical activity (through the DAPHNE sensors, aggregator app, mobile, and Web interfaces); the subjects can also fill in physical activity questionnaires (standardized questionnaires via the Web interface/mobile app) and answer psychological questionnaires (through standardized questionnaires, via mobile and Web interfaces).

3. Physician application:

This is a Web portal and is the interface between the physician and DAPHNE System. It allows physicians to follow-up with their patients in an efficient and innovative way. All the information on a particular patient is centralized in this portal and facilitates the analysis to be performed by the caregiver. This tool also provides options to allow settings for the patient’s physical activity, goals, coaching messages, and so on. Furthermore, the physical activity module also enables sending the subjects educational material promoting a healthier lifestyle.

### DAPHNE Physical Activity and Physiological Sensors

**Figure 3:**
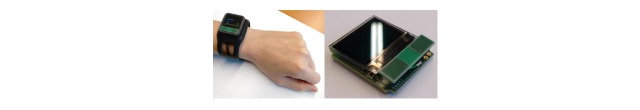
Activity sensor developed in Data-as-a-Service Platform for Healthy Lifestyle and Preventive Medicine (DAPHNE).

Alongside the ICT platform within the DAPHNE project, there is also the development of an innovative physical activity and physiological sensor. There are a number of devices available on the market that measure physical activity behavior. At present, there are a number that would be considered commercial devices and only a few that would be considered “research grade” standard. One of these devices is “Empatica,” which is a wristband that provides raw data from sensors instead of processed signals. This device is able to capture the following parameters at a high acquisition rate: heart rate, skin conductance, temperature, heat flux, and movement. The DAPHNE sensor device is one of several main innovations of the DAPHNE project and it aims to go beyond what is already available. The sensor device pushes the implementation of sensor and communication technology beyond current state of the art technology, by combining many functions (it measures and classifies physical activity, heart rate, and galvanic skin response) within a very small device (Figure 3), for a more precise energy consumption and stress estimation, and by streaming raw measurement data at a very high data rate over low-energy Bluetooth. This enables data to be uploaded automatically and immediately visible in the PHS by both the user and the caregiver.

The sensor has implemented advanced algorithms for the detection of different activities (such as running, walking, lying, or sitting), estimating the energy consumption and measuring stress. The sensor has recently achieved the CE certification for its use in clinical environments. Initial validation studies have been implemented comparing this sensor with other research grade sensors, and also to gold standard measurements of energy expenditure. Results of the validation studies will be presented in further scientific manuscripts.

An aggregator app has also been developed for the connection of the sensor with the Daphne Data Cloud, allowing also the interoperability with other Continua Alliance physical activity sensors.

### DAPHNE Data Cloud

The Daphne data could provide the storage of user data (1) in the cloud, which includes the Public Personal Health Record repository and the Self-Tracking Health Data Repository; and (2) in the hospital facilities, which includes a Personal Health Record repository for each hospital. The separation into 2 different repositories intends to address privacy and security regulations that prevent the user data from leaving the care giver facility. The data cloud manages the data and provides access to user data only to authorized users.

In addition, the user data in the data cloud can be retrieved for secondary use in research as anonymized data sets subject to user consent policy and under preservation of intellectual property rights.

**Figure 4:**
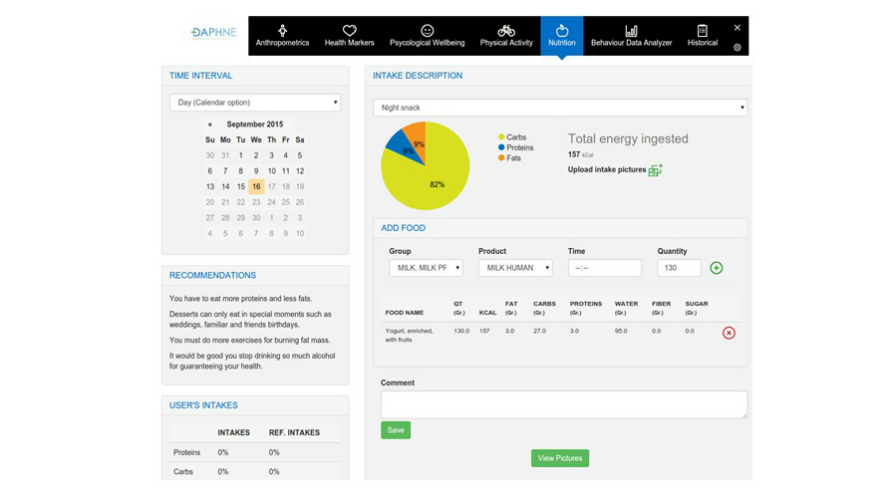
Screenshot of the nutrition section within the Data-as-a-Service Platform for Healthy Lifestyle and Preventive Medicine (DAPHNE) user portal.

### Functional Requirements

The authentication procedures for patients are that they will only be able to register via their physician through the physician portal. On any occasion of use, the PHS will require login details (username and password) to ensure security of data (further details on security are explained later in the manuscript). All users of the system must opt-in and consent to anonymous data analysis (big data processing capabilities) before any data is uploaded to the cloud from the mobile or the user/physician portal; however, they will be able to opt-out at any time. The idea for the anonymous big data processing functionalities is to enable data analytics by the health care and research organizations involved, and perhaps in the long term to share these data with other organizations interested in public health, scientific research, health marketing, or pharmaceutical research.

### Input and Output Data

To achieve semantic interoperability, uniform data representation has been taken into account within the formulation and planning of the DAPHNE system. Terminology systems are used to transform descriptions and values of medical data into universal medical code numbers to achieve an unambiguous understanding of the meaning while the data is transferred from one place to the other. These coding systems (ie, Systematized Nomenclature of Medicine—Clinical Terms, etc) are used for a variety of domains: for example, medicine, public health, and medical informatics.

There are country specific and international classification systems. Internationally endorsed classifications facilitate the storage, retrieval, analysis, and interpretation of data. They also permit the comparison of data within populations over time and between populations at the same point in time as well as the compilation of nationally consistent data.

To address the health information system interoperability needs, DAPHNE will use continuity of care documents (CCD) to export data. CCD is currently one of the most frequently used interoperability standards and it is human-readable using any standard Web browser.

### Anthropometrics and Health Markers

In order to have the most comprehensive view of the user/patients health and behavior, there are a number of categories of input data within the DAPHNE system—anthropometrics, health markers, nutrition, physical activity, and psychological wellbeing. Not all input data will be available for each patient, therefore there is limited compulsory data required and the automatic analysis and recommendations will rely on as little data as possible. The DAPHNE system is configured to provide cut-points based on available clinical guidelines. Firstly, anthropometrics (age, gender, height, weight, waist and hip circumference, fat mass, and fat-free mass) and health markers (blood pressure, cardiovascular fitness, smoking status, fasting glucose, diabetic status, familial history of diabetes, HBA1c, triglyceride levels, medications, and cholesterol levels) can be entered into the PHS. Embedded within the analytics of the DAPHNE system will be the capability of calculating BMI, for example, and then the system will alert the user of their BMI compared with clinical cut points. The same is true for body fatness and a number of health markers. Data can be viewed as a single entry, but also tracked over time periods of 7 days, 14 days, 1 month, and 3 months.

### Nutrition

The user/patients nutrition assessment will be entered and analyzed via the nutrition app designed within the DAPHNE project. An estimation of energy requirements will be calculated using validated equations [30] by the DAPHNE system based on the anthropometric information of the user plus the energy expenditure from the motion sensors. The app works with the European Food Information Resource (EuroFIR) Database [29]. EuroFIR is a member-based international nonprofit association of food composition data compilers, expert users, and stakeholders, based in Belgium. EuroFIR continues in activities established by a successfully completed EuroFIR project, a 5-year Network of Excellence funded by the European Commission’s Research Directorate General under the “Food Quality and Safety Priority” of the Sixth Framework Programme for Research and Technological Development. The EuroFIR database contains food composition information, such as energy, macronutrients (eg, protein, carbohydrate, fat), and their components (eg, sugars, starch, fatty acids), minerals and vitamins of approximately 15,000 food items from different countries within Europe (United Kingdom, Italy, the Netherlands, Spain, Denmark, France, Norway, etc) and the United States. The app will learn from user preferences, facilitating the introduction of food intake to the system. The data required for input includes the food item in grams and the intake description (breakfast, morning snack, lunch, afternoon snack, dinner, evening snack). The app offers the possibility that users can personalize the type of food intake they generally have, adapting to the volumes usually eaten and mixing different foods in personalized meals. Users will be asked to input their food intake after each occasion of consumption of food or drinks. However, in some instances this may not be possible, therefore functionality within the system will enable the user to enter the information retrospectively. In the user portal, the user will then be able to see the analyzed data regarding their dietary intake. For each day, the user can see the nutritional information about his/her food intake. Then, a summary is presented by the system and a complete report is available. In this report, the good behaviors of the user are emphasized and recommendations about bad habits are given. The idea is that the physician and user can manage and track their nutritional behaviors. A special design has been followed for children and adolescents patients with the support of Ospedale Pediatrico Bambino Gesú; (eg, hiding calorie intake information to patients), in order to avoid possible psychological problems in the use of the platform and providing the right type of messages for children and adolescents.

### Physical Activity

The Department of Health (UK) recommended levels of physical activity are at least 150 minutes of moderate intensity physical activity in bouts of 10 minutes or more per week (for example 30 minutes in 5 days or more) [31]. Sedentary behavior has traditionally been used to describe low levels of moderate to vigorous activity. However, the term sedentary behavior from the Latin “sedere” (to sit) has now been categorized as an independent behavior of interest. In this emerging field, sedentary behavior describes a class of activities that have both a low-energy expenditure typically ≤1.5 metabolic equivalent thresholds, and a sitting or reclining position [32-34]. “Inactivity,” on the other hand, should be used to describe those who are not performing sufficient amounts of moderate to vigorous physical activity [35]. There is building evidence that this sedentary behavior is adversely associated with a number of health markers, some relationships seen across the lifespan. The types and amount of sedentary behavior engaged in may be different in children compared with adults due to differences in how time is spent and choices made about leisure time versus obligatory activity [36], therefore it is important to consider these separately. It has also been demonstrated that sedentary behaviors increase and that physical activity decreases during adolescence [37], and this development of early sedentary behaviors may form the foundation for such behaviors in the future [38]. In this input category, sedentary behavior, physical inactivity, and physical activity will be categorized. Input data will be automatically uploaded from the DAPHNE sensor and aggregator providing information on energy expenditure, time in different intensities of activity, mode of activity, and frequency of activity performed. Energy expenditure will be calculated for the whole day and also activity energy expenditure (ie, removing energy expended during sedentary behavior). Time in sedentary behavior, light, moderate, and vigorous intensity activities will be categorized during waking hours. The type of activity will also be recognized (lying, sitting, walking, running, etc). These input data will be compared with the recommended physical activity guidelines and users will be advised on their current status (meeting recommendations for physical activity or physically inactive) and also be provided with recommendations on how to improve (eg, reducing periods of sedentary time). Both daily and 7-day average activity pattern will be inputted into the DAPHNE system as an indicator of time spent in sedentary behavior and physical activity. People do not always perform the same amount of activity every day and weekdays may differ markedly to weekend days. The 7-day average is therefore a better indicator of lifestyle than 1 day on its own. Furthermore, users will have the option to add activities manually into the user portal in the case that physical activity devices are not worn. The physical activity module will enable caregivers to devise treatments plans for their patients, which include type and intensity of activity, time of activity, scheduling of activities, setting reminders, goals for duration, intensity, target heart rate, and so on. These programs are accessible by either mobile app or Web interface, allowing the patients to make minor adjustments to the training program, for instance choosing between activity types of the same intensity level. The physical activity module will also provide the user with an indication of hisher performance compared with the goal set by the care provider. The patient will receive reports from the system of his/hers physical activity. Those reports will have the option to be filtered and sorted, in order to provide the patient a tool to see his/her progress in physical status. If motion sensors are not available, physical activity will be monitored using validated questionnaires within the DAPHNE project using the physical activity module of the system.

### Psychological Wellbeing

The DAPHNE system will be designed to capture assessments of psychological wellbeing via an app, which will incorporate many validated questionnaires to assess constructs, such as anxiety, stress, depression, and quality of life, among others. The DAPHNE system will be set up so that the physician can choose from a large library of validated questionnaires; the user will then receive a notification that the physician has requested they fill in particular questionnaires, which they can do within the psychological wellbeing app. The scoring outcomes have been automatically incorporated into the DAPHNE system so that the physician will be alerted to unfavorable scores on the questionnaires and will be able to deal with those in the correct manner. Again, scores can be tracked over time, but due to the nature of the questionnaires measuring trait characteristics of the person, it is unlikely that the questionnaires will be completed more than once per month. Furthermore, there will be an added functionality within the psychological wellbeing app where the user can type in journal entries, which can be seen by the physician.

### Behavior

The final segment of the user portal will allow the user/patient to see an overview of their behavior—in terms of the food consumption and physical activity. Here, they will be able to view their physical activity in amounts of sedentary, light, moderate, and vigorous physical activity segments that have accumulated across the day alongside the accumulation of calories, fat, carbohydrate, and protein. Recommendations to increase the levels and intensity of activities performed and decrease intake of unhealthy foods will be sent automatically. In the case of patients, recommendations will only be sent after the approval of the physician. Physicians will have the capacity to provide personalized recommendations to the patient through the DAPHNE system. Users will be able to view data for a single day, or accumulated over 7 days, 14 days, 1 month, and 3 months in order to track their behavior change over time.

### Privacy and Security

Due to the sensitive nature of the data used/processed by the DAPHNE project it was imperative to take into account the EU Data Protection and Electronic Communications Directives to comply with current EU data protection regulations; and further meet current General Data Protection Regulation principles.

Therefore, the Consortium adopted the use of the Privacy by Design (PbD) and Security by Design (SbD) paradigms into the development of the DAPHNE project. These paradigms incorporate privacy and security aspects from the earliest stage of the project and they can be defined as follows: (1) PbD is an approach to system engineering, which takes privacy into account throughout the whole engineering process; and (2) SbD is an approach to system engineering where measures to protect ICT assets have been designed throughout the whole engineering process.

**Figure 5:**
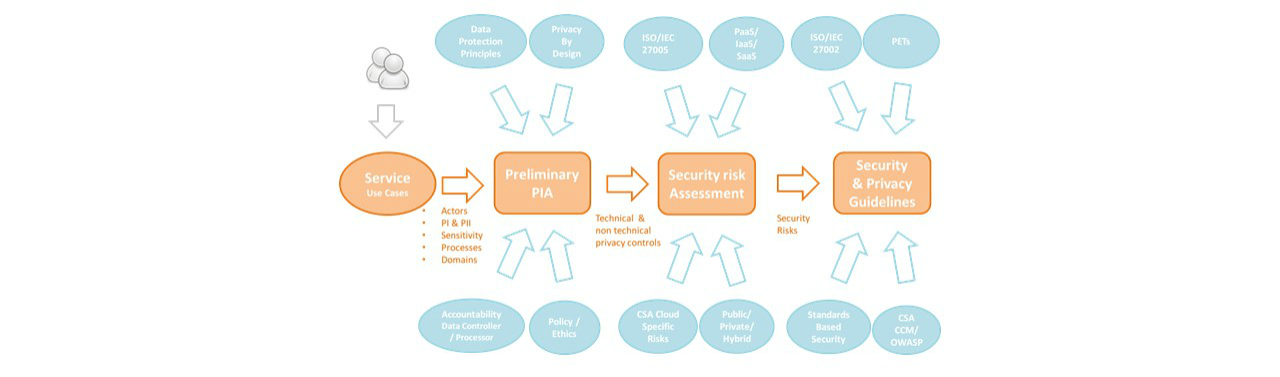
Methodology to be used to achieve privacy and security in the Data-as-a-Service Platform for Healthy Lifestyle and Preventive Medicine (DAPHNE) project.

The methodology used to achieve Privacy and Security by Default paradigms is shown in Figure 5.

There were 4 steps followed: (1) define the service and identify personal data collection, processing, and sharing; (2) early in the project, carry out a Preliminary Privacy Impact Assessment with respect to EU Data Protection Principles and the organization’s own data and ethical policies; (3) perform a risk assessment for the cloud architecture to be deployed; and (4) define the security controls and privacy guidelines for the system.

### Awareness

The majority of the security control mechanisms used within DAPHNE are imposed in order to comply with actual legislation. However, the awareness services go one step beyond the current legal constraints. The main aim of the awareness services is to keep the patient informed of the access made to their sensitive data. In order to do so, the system will track and audit every single access to the patient information. These services will provide them with a list of the different occasions of access, specifying who has made access, from which group or role, and the time of each access.

## Results

The project has been funded and the research work has started. Results for the validation of the different components are due in March 2017.

## Discussion

### Overview of Progress

The aim of this paper was to describe and detail the structure of the DAPHNE ICT platform and fully describe the data that is required to be inputted into the system and the data that will be available to the user. The DAPHNE project will provide personalized ICT services for the prevention of overweight and obesity, taking into account anthropometry, health markers, food intake, physical activity, and psychological wellbeing. To our knowledge, this system will be the most holistic approach toward overweight and obesity in that it incorporates features from these different sections and allows them to be tracked and visualized simultaneously. Personalized sensors, models, and services will be developed, based on clinical requirements to increase physical activity, reduce sedentariness, and improve eating habits in order to initiate behavior change in a beneficial way for weight loss and maintenance. A particularly innovative feature of the DAPHNE system is that it is designed for use in clinical settings, providing an additional link between physicians and patients that does not require further appointments or face-to-face time. As a difference with previous existing solutions, the DAPHNE project tackles the obesity problem from a clinical point of view, designing the different interfaces for its use by patients (adults and children), physicians, and caregivers. Interoperability with existing medical systems and clinical standards has been taken into account, for its future deployment in public and private hospitals in Europe and worldwide.

### Validation Procedures

There are several steps of validation that are planned as part of the DAPHNE project over the coming months and years. Firstly, the components of the system have to be validated to ensure they measure what they are supposed to. For example, the physical activity sensor will be validated against other highly validated devices and against gold standard indirect calorimetry measures of energy expenditure. Additionally, it must be ensured that all components of the system work together correctly, for example, the different mobile apps being developed must all link and show data consistently and correctly within the PHS part of the system. Initial feedback regarding the degree of motivation of the end users in the usability and feasibility of the system will be sought and evaluated at a primary endpoint of the DAPHNE project. Once the short-term studies are finalized in these areas and any problems are resolved by the technical partners in the project, the longer term objectives of DAPHNE will be studied. These include a clinical validation of the system in different European Hospitals (Ospedale Pediatrico Bambino Gesù [OPBG], Italy, and Maccabi Healthcare, Israel) with different end users in mind: (1) in OPBG, the system will be used by adolescents with obesity problems, under the supervision of their parents; and (2) in Maccabi, the system will be used by adults with obesity problems.

Both clinical trials will be conducted over 12 weeks and will compare the usual care provided to these obesity patients with the usual care plus the addition of the DAPHNE platform and physical activity sensor. Alongside comparison of the overall results in terms of clinical outcomes (markers of weight, body composition, and health), there will also be feasibility and usability questionnaires about the system in order to assess the patients’ views of the platform. The planning for this project is detailed and strong with the consortium working hard in the funding process and planning stages of the project. We are looking forward to progressing through the different stages of the project and to publishing the details and results in the near future.

## Abbreviations

BMI: body mass index
CCD: continuity of care documents
CVD: cardiovascular disease
DAPHNE: Data-as-a-Service Platform for Healthy Lifestyle and Preventive Medicine
EuroFIR: European food information resource
OPBG: Ospedale Pediatrico Bambino Gesù
PbD: privacy by design
PHS: personal health services
SbD: security by design
